# Role of Plant-Derived Smoke Solution on Plants Under Stress

**DOI:** 10.3390/ijms26167911

**Published:** 2025-08-16

**Authors:** Amana Khatoon, Muhammad Mudasar Aslam, Setsuko Komatsu

**Affiliations:** 1 Department of Botany, Kohat University of Science and Technology, Kohat 26000, Pakistan; amanakhatoon@kust.edu.pk; 2 Department of Botany, University of Science and Technology, Bannu 28100, Pakistan; mudasaraslam@ustb.edu.pk; 3 Faculty of Environment and Information Sciences, Fukui University of Technology, Fukui 910-8505, Japan

**Keywords:** plant-derived smoke solution, plant, seed germination, plant growth, stress tolerance

## Abstract

Plants are constantly exposed to various environmental challenges, such as drought, flooding, heavy metal toxicity, and pathogen attacks. To cope with these stresses, they employ several adaptive strategies. This review highlights the potential of plant-derived smoke (PDS) solution as a natural biostimulant for improving plant health and resilience, contributing to both crop productivity and ecological restoration under abiotic and biotic stress conditions. Mitigating effects of PDS solution against various stresses were observed at morphological, physiological, and molecular levels in plants. PDS solution application involves strengthening the cell membrane by minimizing electrolyte leakage, which enhances cell membrane stability and stomatal conductance. The increased reactive-oxygen species were managed by the activation of the antioxidant system including ascorbate peroxidase, superoxide dismutase, and catalase to meet oxidative damage caused by challenging conditions imposed by flooding, drought, and heavy metal stress. PDS solution along with other by-products of fire, such as charred organic matter and ash, can enrich the soil by slightly increasing its pH and improving nutrient availability. Additionally, some studies indicated that PDS solution may influence phytohormonal pathways, particularly auxins and gibberellic acids, which can contribute to root development and enhance symbiotic interactions with soil microbes, including mycorrhizal fungi. These combined effects may support overall plant growth, though the extent of PDS contribution may vary depending on species and environmental conditions. This boost in plant growth contributes to protecting the plants against pathogens, which shows the role of PDS in enduring biotic stress. Collectively, PDS solution mitigates stress tolerance in plants via multifaceted changes, including the regulation of physico-chemical responses, enhancement of the antioxidant system, modulation of heavy metal speciation, and key adjustments of photosynthesis, respiration, cell membrane transport, and the antioxidant system at genomic/proteomic levels. This review focuses on the role of PDS solution in fortifying plants against environmental stresses. It is suggested that PDS solution, which already has been determined to be a biostimulant, has potential for the revival of plant growth and soil ecosystem under abiotic and biotic stresses.

## 1. Introduction

Fire has been a part of the ecosystem process ever since plants colonized land millions of years ago. Wildfires are one of the most serious natural hazards affecting widespread plant ecosystems around the world [1]. The degree to which vegetation is impacted depends on the frequency, severity, extent, and intensity of fire [2]. Ecological processes such as seed germination, seedling growth, flowering, and fruiting are boosted with fire in certain ecosystems such as boreal forests and Mediterranean scrub [3,4]. These boosting effects may arise as a result of various mechanisms including the removal of competing vegetation, the release of nutrients, and the breaking of seed dormancy. Some plant species, such as *Banksia* spp. (Australia), *Pinus* spp. (North America), and *Pelargonium* spp. (South Africa), have evolved to depend on fire cues, particularly heat or smoke, to break seed dormancy and initiate germination by cracking or weakening the seed coat. Due to increasing interaction between urban areas and natural ecosystems by the intensifying effects of climate change, the frequency, severity, and extent of fire have been rising worldwide, often generating adverse effects, even in fire-adapted ecosystems [5,6]. Fire products include heat, chemicals, ash, and smoke, which are a complex mixture of biologically active compounds such as karrikins (KARs), butenolides, cyanohydrins, and nitrates. All these components can have both positive and negative effects on plants depending on the species, developmental stage, exposure duration, and environmental conditions. While smoke has been shown to have hazardous effects on plants and other living organisms, its role shifts to a positive entity when used in solution form by bubbling into water to form plant-derived smoke (PDS) solution. Based on this potential, smoke was widely identified as germination cues for different species from both fire-prone and fire-free ecosystems [7]. The positive effects of fire and fire products are associated with the optimum requirements of plant species for these components. It is therefore assumed that plant communities are affected varyingly by fire and fire products.

PDS generated during fire is a well-known agent for promoting plant growth and development, which positively affects plant species from different habitats [8,9]. PDS is obtained from burning different parts of plants including shoot, leaves, and straw or whole plant [10]. Species related to a fire-prone environment positively responded to smoke produced during forest fire [11]. The promotive effects of PDS are independent of seed size, shape, and type [12]. PDS solution enhanced germination process in plant communities of climatically diverse regions including South African Mediterranean [13] and Californian chaparral [11]. PDS solution is a promising factor for several growth-related phenomena of plants including breaking seed dormancy and accelerating seed germination [10]. PDS solution promoted seed germination in wheat (*Triticum aestivum* L.), maize (*Zea mays* L.), soybean (*Glycine max* L.), Arabidopsis (*Arabidopsis thaliana* L.), and lettuce (*Lactuca sativa* L.) by increasing the activity of hydrolytic enzymes, which supports the mobilization of stored food reserves [12]. PDS induced many changes in seeds, including the sensitivity of seeds to phytohormones [14] and permeability by softening the seed coat [15]. This evidence confirmed that the efficacy spectrum of PDS solution is quite diverse and follows different pathways to affect the germination processes.

Positive effects of PDS solution at germination and post-germination levels were observed in crops, such as rice (*Oryza sativa* L.) [16], wheat [17], and maize [18]. PDS solution treatments improved growth and yield in okra (*Abelmoschus esculentus* L.) [19]. It has positive effects on growth attributes such as root/shoot length, seedling fresh/dry biomass, lateral roots, and adventitious roots of acacia (*Vachellia hebeclada* DC.) [20], maize [21], ipomoea (*Ipomoea batatas* L.) cuttings [22], and members of the Amaryllidaceae family [23]. PDS positively affected the post-germination growth of rice [24], chickpea (*Cicer arietinum* L.) [25], soybean [26,27,28,29], and wheat [30,31]. Studies on the post-germination of crops treated with the PDS solution elucidated that its treatment affected not only the seed germination stage but also the plant growth and development stages. These findings suggest that PDS solution has a perpetuating impact on the plants, extending beyond the immediate period of germination.

PDS solution enhanced growth attributes in different plant species. Photosynthetic pigments, like chlorophyll contents and carotenoids, total nitrogen, total soluble proteins, and photosynthetic rates increased in rice with PDS treatments [24]. Total soluble sugar, α-amylase activity, starch, protein contents, lipase activity, and lipid contents increased in lettuce treated with PDS solution [32]. Photosynthetic pigments, total phenolics, total flavonoids, and proanthocyanidins increased in *Tulbaghia* species treated with PDS solution [33]. PDS has positive effects on nutrient uptake, including nitrogen as well as zinc, iron, and copper ions in papaya (*Carica papaya* L.) seedlings [34]. These results suggest that PDS solutions are equally effective to improve physiological attributes in plants.

Abiotic stresses such as salinity, flooding, drought, heavy metals, and temperature stress are serious threats to plant growth and yield [35]. Plants have established numerous morphological, physiological, and cellular defense mechanisms to protect, avoid, or tolerate the adverse effects of abiotic stress [36]; however, not all plant species are able to modify their responses to cope with these stressors. In this regard, plants need various measures to sustain life in such conditions, and PDS solutions are the best natural approach. PDS application reduces the drastic effects of abiotic stress on plants. Growth retarding effects of salt stress in maize seedlings were reported to be minimized by PDS solution application via activating ascorbate peroxidase (APX) and peroxidase (POD) enzymes [37]. Lipid peroxidase contents were reported to drop significantly in wheat seedlings treated with PDS solution in the presence of salt stress, suggesting that PDS solution plays an important role in reactive-oxygen species (ROS) scavenging [38]. PDS solution acts on proteins to confer stress tolerance in soybeans against both flood and salt stress [29,31]. PDS application decreases proline contents, while improving photosynthetic pigments and soluble sugar in lead (Pb)-stressed rice seedlings [39]. These findings provide clues as to how PDS solutions can improve plant growth and yield loss. This review focuses on the mitigating effects of PDS solution on stressed plants while considering a broad spectrum of morphological, physiological, and molecular responses.

## 2. Chemical Nature of PDS

PDS solution is a complex mixture of compounds derived from burning a natural mixture of plant materials and stimulates seed germination and plant growth from a wide range of plants [40]. Recent literature has proved that KARs are the active components responsible for PDS solution-induced activities [40]. KAR acts as a protective agent against abiotic stresses such as drought, salinity, and extreme temperature by regulating plant adaptation to these stresses. The active compounds present in PDS solution were isolated from a variety of plants, indicating that the properties of the smoke solution vary depending on the type of plant producing the smoke. Different compounds isolated from PDS solution are mentioned in Table 1.

### 2.1. Karrikins

PDS solution is the source of different active and passive compounds. This diverse chemistry of PDS solution might be the reason behind its broad spectral impact on different growth responses of plants. PDS solution contains thousands of active compounds to promote the growth of different plants [58]. KAR is a small class of signaling molecules, which belong to the butenolide family. Structurally, KAR consists of a five-membered butenolide ring fused to a six-membered pyran ring [59]. KAR interacted with plant phytohormones and unknown transcription factors like proteins to process endogenous signals for dormancy breakdown and seedling vigor process [60]. These results further support that compounds in PDS solution are stable and diverse, which affects the growth processes in several ways.

The literature revealed that KARs play a key role in different biological processes, including seed dormancy release, germination regulation, and seedling establishment. KAR1, at very low concentrations (around 10^−9^ M), can work as a seed primer agent and stimulate seed germination [61]. KARs induce seed germination by interacting with phytohormones like gibberellic acid (GA) and abscisic acid (ABA) [62]. Interestingly, KAR1 regulates some light-induced genes, such as *GA3oxs* and *YUCCA* [62]. In addition, KAR1 can also interact with nitrogen oxide to induce seed germination in wild oat by stimulating the production of ethylene. This stimulation occurs through the activation of two enzymes involved in ethylene biosynthesis: 1-aminocyclopropane-1-carboxylic acid (ACC) synthase (ACS) and ACC oxidase (ACO) [46]. The ability of KARs to enhance seed germination has significant practical applications in crops. KARs can be used to improve seed germination and growth promotion in crops such as wheat, maize, and rice, particularly in species that exhibit high dormancy.

The KAR signaling pathway impacts root hair growth, lateral root proliferation, and nodule formation [63,64]. KAR signal transduction promotes root hair elongation by affecting auxin and ethylene [65]. KAI2 controls the accumulation of Auxin Transporter Protein 1 (AUX1) and Pin-Formed 2 (PIN2), inhibits the expression of 1-Aminocyclo-Propane-1-Carboxylate Synthase 7 (ACS7), downregulates ethylene synthesis, and promotes root hair elongation under low levels of exogenous phosphorus [66]. For lateral root formation, D14 and KAI2 coregulate lateral root density, which differs from other root traits in Arabidopsis [67]. KAR signaling positively regulates the synthesis of auxin and jasmonic acid while negatively regulating ABA synthesis, ultimately leading to the promotion of soybean root nodule formation [63]. By enhancing root hair elongation and lateral root formation, KARs can improve nutrient uptake efficiency, particularly under nutrient-limited conditions. This regulatory function of KAR can be harnessed to develop crops with improved root growth performance.

### 2.2. Other Components

Besides butenolides, many other growth regulating compounds were identified in PDS solution, most of which have positive effects on germination and plant growth. Cyanohydrin is one of the major seed germination stimulants isolated from PDS. Several related cyanohydrins such as mandelonitrile, acetone cyanohydrin, glycolonitrile, and 2, 3, 4-trihydroxy-butyronitrile are present and stimulate the seed germination of different plant species. The activities of these compounds are due to the spontaneous release of cyanide, suggesting an ecological role for cyanide in the post-fire revival of plant communities [54]. Cyanide has been reported to alleviate seed dormancy by stimulating ROS generation, which act as signaling molecules for germination. It was suggested that cyanide signaling involves the inhibition of enzymes including catalase and superoxide dismutase and their effect was linked with an obvious increase in ROS including hydrogen peroxide and superoxide anion generation [68]. This enhanced ROS production and signaling finally causes improved seed germination. The novelty of discovering cyanohydrins in smoke solution confirms cyanide as an important germination stimulant in post-fire environments [54]. Because cyanohydrins stayed for a long time in the top layers of the soil where most of the dormant seeds are present, they are readily available as a growth-promoting signal [54]. This is further strengthened by the fact that chemical compounds must be added and left in the soil for an appropriate period to be effective in a post-fire environment.

Catechol is another major component of PDS and induces root growth and suppresses root hair elongation of tobacco through ROS-mediated redox signaling [55]. Catechol is a phenolic compound that plants produce through the shikimate pathway [69]. Catechol compounds are mainly produced by plants for their growth, development, and protection [70]. Catechol is a widely distributed chemical in the natural environment and was discovered by destructive distillation of the plant extract catechin (argan oil) [71]. Catechols are produced through the metabolism of cholesterol by bacteria such as *Mycobacterium tuberculosis* [72]. Catechol is associated with the enzyme activities of the oxidation–reduction pathway, such as catalase (CAT), POD, superoxide dismutase (SOD), and other antioxidant systems [73]. The role of catechols in plant growth is multifaceted, which involves interaction with different phenomena including growth, biochemical, and hormonal signal regulation in plants.

PDS solution contained 3,4,5-trimethyl-2(5H)-furanone or tri-methyle butenolides (TMB), which inhibited seed germination [51]. These inhibitory compounds, 5,5-dimethylfuran-2(5H)-one and (5RS)-5-ethylfuran-2(5H)-one, are isolated from skilpadbessie (*Passerina vulgaris* Thoday) and red oat grass (*Themeda triandra* L.)-derived smoke solution, respectively [53]. Based on the structural similarity of TMB to KAR, at first, it was hypothesized that TMB interferes with KAR signaling; however, later on, physiological and molecular analyses implicated that TMB exerts its germination inhibitory effect independently of KAR signaling [74]. These results support the statement that KAR and TMB have independent pathways to regulate seed germination. Komatsu et al. [31] reported that PDS solution improved the growth of wheat seedlings under salt stress. This stress alleviation was proposed to involve the regulation of energy metabolism and the ubiquitin–proteasome system. They concluded that the alleviation of salt stress by PDS solution largely involves the regulation of ascorbate peroxidase, ubiquitin, and H^+^-ATPase in the growth inhibition caused by salt stress. Using metabolomic and proteomic analyses, key metabolites and proteins associated with these pathways were identified, suggesting that PDS solution enhances wheat’s ability to cope with the negative effects of salt stress. Further research in line with this study is needed to have a clear and deep understanding of PDS-mediated stress alleviation mechanisms.

## 3. Effects of PDS on Seed Germination Parameters

**Figure 1:**
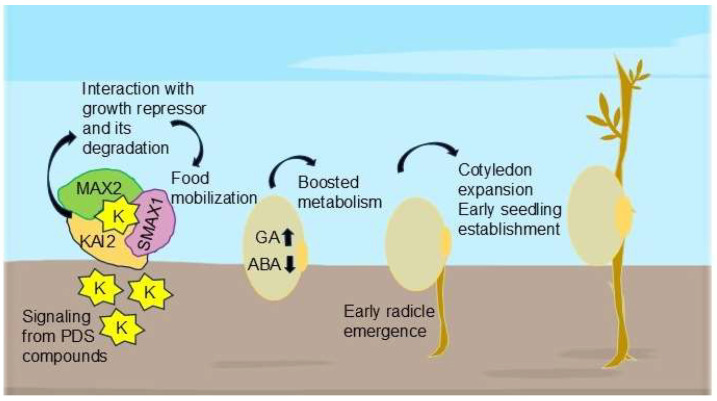
The accelerating effects of PDS solution on seed germination. PDS-derived karrikins (K) interact with an α/ß-hydrolase (KAI2) and then with MAX2 to degrade growth-suppressing proteins. This signals a hormonal shift, causing the regulation of the GA and ABA balance, which mobilizes the stored starch in seeds and finally accelerates seed germination. The upward and downward arrows represent the increase and decrease, respectively.

After the discovery of PDS as a plant growth regulator, most of the research has been conducted and reported on the morphological and physiological aspects of seed germination. Seed germination is a complex process where the seed undergoes several changes including recovery from maturation drying; starting metabolic operations; completing critical cellular events that allow the embryo to emerge; and preparing for seedling growth [75]. Many plant species respond to the PDS solution, but not to the same extent [76]. Smoke solution positively affected the seed germination of fire-free and fire-prone habitat plant species and also many crops [77,78]. PDS and the derived compounds have been reported to stimulate seed germination in different plants (Figure 1; Table 2).

### 3.1. Effect of PDS Solutions on Germination in Arabidopsis

Arabidopsis is an important species for investigating plant responses to environmental stress. In Arabidopsis, like any other plant, adverse environmental conditions affect growth and development by impairing water intake, osmotic imbalance, and ion toxicity, ultimately decreasing seed germination, seedling establishment, and overall plant growth [90]. PDS has emerged as a promising strategy for increasing germination and stress tolerance. According to research, KAR improves seed germination by increasing GA production while decreasing ABA buildup, encouraging early seedling growth [91]. Among the well-studied smoke-derived chemicals, KARs have been found to stimulate seed germination and seedling photomorphogenesis in Arabidopsis by interacting with critical regulatory mechanisms.

The KAR signaling pathway includes the Karrikin Insensitive 2 (KAI2) receptor and the F-box protein More Axillary Growth 2 (MAX2), which also regulates strigolactone (SL) signaling. Further research has shown that KARs interact with auxin transport pathways, resulting in improved root and shoot growth under stress conditions [42]. KARs reduced dormancy in numerous plant species by suppressing dormancy-related genes and regulating hormonal signaling pathways such as ABA and GA [63]. This evidence pointed out a possible correlation of KARs with phytohormones, which coherently interact to regulate physiological function optimally.

Furthermore, biochemical experiments show that PDS-treated Arabidopsis plants have significantly lower levels of lipid peroxidation, bolstering its protective action against oxidative stress [92]. PDS treatment increases potassium absorption while decreasing sodium ion buildup in Arabidopsis, optimizing ion homeostasis during saline stress [93]. This suggests that plants have developed unique biochemical pathways for interpreting and responding to different smoke-derived signals, allowing them to maximize germination time under changing environmental conditions. This difference in response to PDS solution components highlights the complexities of seed dormancy regulation and the need for more research to fully understand the functional linkage between smoke-derived bioactive chemicals and seed germinating genes in Arabidopsis [94]. According to experimental findings, prolonged exposure to TMB has been observed to slow radicle protrusion and enhance dormancy in Arabidopsis seeds. However, not all smoke-derived compounds promote germination. TMB, which is a furanone molecule present in PDS, suppresses germination in Arabidopsis by influencing hormonal signaling pathways and delaying seed activation.

TMB-treated Arabidopsis seeds exhibit physiological changes associated with dormancy maintenance, highlighting the complexity of smoke-derived compounds in seed germination regulation [74]. These investigations underscore the diverse roles of PDS components in Arabidopsis and emphasize the need for further research to explore their potential applications in seed biology and stress adaptation [74]. Furthermore, Arabidopsis seeds with impaired ABA biosynthesis exhibit reduced sensitivity to TMB, reinforcing its role in dormancy induction [74]. Comparative investigations also suggest that TMB-treated Arabidopsis seeds experience lower water uptake during inhibition, further supporting its inhibitory effect on germination [74]. These findings highlight the intricate link between smoke-derived compounds and seed dormancy mechanisms, emphasizing the need for further research on their potential applications in seed germination.

### 3.2. Effects of PDS Solutions on Germination in Other Plants

PDS solution produced by burning plant parts such as the leaf, shoot, and stem facilitates the seed germination and plant growth processes of over 1200 different plant species belonging to 80 different genera [76]. Regarding plant life cycle, PDS enhanced the seed germination of celery (*Apium graveolens* L.) and maize [18]. PDS enhanced the seed germination of maize [95] and released seed dormancy in rice [96]. PDS solution enhanced seed germination in plants including chickpea [25], pea [97], soybean [27], maize [98], and apricot seeds [93]. It is concluded that PDS is highly diverse in its efficacy as the majority of plant species positively respond to it in the enhancement of seed germination. PDS solution showed maximum enhancement of seed germination in plant species [13]. PDS solution significantly promoted seed germination in Arabidopsis and lettuce by increasing the activity of hydrolytic enzymes including amylase, lipase, and protease, which are essential for converting stored starch, lipids, and proteins into usable energy for the emerging embryo. These results explain the way PDS solution boosts the seed germination process of different plants via triggering the energy production process.

## 4. Effects of PDS on Plant Growth Parameters

**Figure 2:**
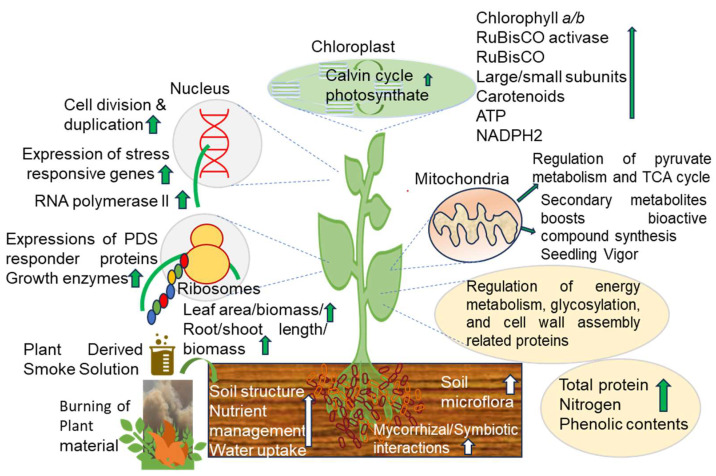
The growth-promoting effects of PDS solution via the modulation of physio-chemical processes. Growth promotion via PDS solution involves the regulation of morphological-, physiological-, and molecular-level responses. PDS solution triggers the expression and accumulation of stress-responsive genes and proteins, which regulate photosynthesis and energy metabolism. Soil structure, nutrient management, and microbial communities are improved in response to PDS solution promoting soil health, which positively contributes to plant growth. The upward arrows represent the increase in the marked phenomena.

After the authentication of PDS as an important germination cue and a growth regulator in different plant growth-regulated phenomena, the effects of PDS solution at the post-germination level were observed in crops, such as rice [99], pea [97], wheat [31], maize [100], and soybean [28,29]. The positive effects of PDS on the morphological and physiological growth of many plants are reported (Figure 2; Table 3).

### 4.1. Effects of PDS Solution on Morphological Growth Attributes of Plant Growth

PDS solution can positively affect plant growth and development at various stages. The positive effects of PDS solution are not dependent on seed size, plant species, genera, and families belonging to gymnosperms and angiosperms [115], commercial crops, and different medicinal plants [87]. Enhanced seedling length and mass in response to PDS solution supports its role in the increased crop yield of different plants [15]. Sreekissoon et al. [116] reported that PDS solution application increased seedling growth in kanna (*Sceletium tortuosum* L.). Soybean treated with PDS solution developed better root and hypocotyl length/weight [29]. Similarly, PDS solution application enhanced wheat growth [31]. PDS solution for the root length, shoot length, and seedling weight of rice was shown to have a positive effect [99]. These studies emphasize the effects of PDS solution on improving multi-dimensional growth aspects in various plant species.

PDS has a positive influence on the seedling growth of several plants including capsicum (*Capsicum annuum* L.), salvia (*Salvia officinalis* L.) [117], maize [18], rice [16], chickpea [25], sorghum (*Sorghum bicolor* L.) [118], soybean [26,28], and wheat [31]. PDS solution enhanced the number of secondary roots, number of leaves, and seedling length/weight in pea seedlings [97]. Soybean root length/hypocotyl length/fresh weight [26,27,28] and seedling fresh/dry weight in cabbage [112] was significantly increased by PDS treatment. A high concentration of smoke solution had inhibitory effects on plant seedling length, while seed germination and seedling growth were boosted when lower concentrations of PDS solution were used [15]. The compounds present in PDS also enhanced seedling growth in different plant species. KAR application improved the survival rate, shoot/length, seedling fresh weight, lateral roots length, and number of total lateral roots in popcorn plant [80]. These results highlight the positive role of PDS solutions on the growth of plant species belonging to different genera and families.

### 4.2. Effects of PDS Solution on Physiological and Biochemical Growth Attributes of Plants

PDS solution enhanced biochemical growth attributes in different plant species. Photosynthetic pigments, such as chlorophyll contents, total nitrogen, total soluble proteins, and photosynthetic rates, increased under PDS solution treatments [24]. Total soluble sugar, α-amylase activity, starch, protein contents, lipase activity, and lipid contents increased in lettuce plants treated with PDS solution [32]. Photosynthetic pigments, total phenolics, total flavonoids, and proanthocyanidins increased in *Tulbaghia* species treated with PDS solution [33]. Aslam et al. [18] confirmed the positive effects of PDS solution on photosynthetic pigments, carotenoid contents, and total soluble proteins in maize leaves. PDS solution has been determined to enhance the root system and positively affect physiological parameters including photosynthetic pigments, nitrate contents, total flavonoids, total soluble sugar, and the level of antioxidant enzymes in chickpea [25], wheat [17], and maize [37,119]. These reports clarified that stimulatory effects of PDS solution perpetuate in the post-germination stage equally, thus resulting in enhanced growth, pigments, and finally increased biomass of the plants.

The application of PDS solution increased calcium ions, potassium ions, nitrogen, protein contents, and total soluble sugar in maize seedlings [37]. The application of PDS solution enhanced the activity of α-amylase enzyme in cockspur grass (*Echinochloa crus-galli* L.) seeds [120]. PDS treatment increased the activities of ribulose 5-bisphosphate carboxylase/oxygenase (RuBisCO) small subunit/large subunit chlorophyll *a*/*b* contents in wheat leaves [30]. An increase in ATP abundance and ATP contents in soybean treated with PDS solution was also observed [121]. PDS solution improved the chlorophyll content, stomatal conductance, breakdown of lipids, and sesquiterpene biosynthesis pathway in grapevine (*Vitis vinifera* L.) [122,123]. In combination with plant growth-promoting rhizobia, PDS solution promoted chlorophyll contents, electrolyte uptake, total soluble proteins, total soluble solids, and POD activities in rice [124]. The application of PDS solution enhanced the biochemical and physiological growth parameters in tomato [109]. Total chlorophyll contents, stomatal conductance, net photosynthetic rate, and β carotenoid contents increased in carrot seedlings treated with PDS solution and KAR [111]. The possible growth triggering connection between PDS solution and soil microflora can be inferred, which further supports plant growth and development.

The contents of chlorophyll, total phenolic, and total flavonoid were increased in banana (*Musa acuminata* Colla.) by PDS solution and KAR treatments [33,34]. KAR positively affects leaf water contents, stomatal conductance, photosynthetic rate, photosynthetic pigments, membrane stability, leaf water potential, and soluble sugar contents in cabbage [112]. Nitric oxide contents increased in soybean roots treated with PDS solution [27]. PDS treatment enhanced photosynthetic pigments, total soluble sugar, and total soluble proteins in pea seedlings [97]. These findings concluded that growth improvement by PDS implies the regulation of various biochemical and physiological attributes in plants.

## 5. Effects of PDS Solution on Plant Stress Tolerance

**Figure 3:**
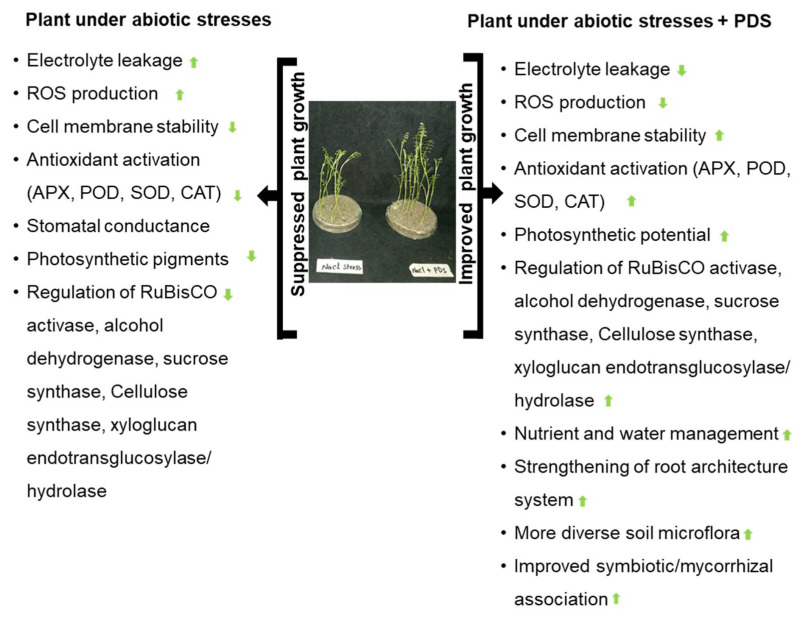
Improving plant resilience and stress adaptation by PDS solution. Overview of abiotic stress effects on plants and PDS solution mechanisms involved in stress tolerance. Abiotic stress negatively affects plant growth and development. PDS solution, through regulation of morpho-physiological- and molecular-level processes, can increase plant stress tolerance and alleviate growth suppression in plants. The upward and downward arrows represent the increase and decrease in the marked phenomena, respectively.

The negative impact of abiotic stress on plants has been observed from morphological to molecular levels and is visible in all phases of plant development [30,125]. The ameliorative role of PDS solution against detrimental effects of salinity, heavy metal toxicity, and flooding stress has been recognized in crops such as rice [39], soybean [26,27], and wheat [17,38,126,127]. Recent research has shown that PDS solution can also modulate abiotic stress responses in Arabidopsis, including better germination rates, antioxidant enzyme activity, and osmotic balance under drought and salt stress conditions [128]. KAR was reported to be involved in stomatal closure, the regulation of cuticle formation, membrane integrity, and anthocyanin biosynthesis, which contributes to the alleviation of osmotic stress in Arabidopsis [129]. Furthermore, a gene expression study verified that smoke-treated Arabidopsis plants demonstrated significantly changed expression levels of genes related to photosynthesis and the metabolism of trehalose and glucosinolates [130]. These results suggested an optimum regulation of physiological functions by the coordinated expression of genes and proteins, resulting in increased abiotic stress tolerance. However, the is no direct literature available that mentions the ameliorating effects of PDS solution on biotic stress but a lot of publications are available that highlight the altering of those chemicals, enzymes, or pathways by PDS solution application, which reduces biotic stress. In this section of the review, PDS-induced stress tolerance in plants under abiotic stress is highlighted (Figure 3, Table 4).

### 5.1. Biotic Stress Resistance

Plants face biotic stress challenges such as bacteria, fungi, viruses, parasites, insects, and weeds, which have a significant negative impact on plant growth and ultimately decrease the quantity and quality of plant yield [132]. Plants are exposed to several biotic stresses and unfavorable environmental conditions that can affect their morphological, biochemical, and molecular processes [133]. Climate change has brought with it a major challenge, as many minor biotic stresses are now becoming major ones and, therefore, continuous modification and integration of existing strategies are extremely crucial to achieve effective crop protection [134]. Biotic stress can lead to heavy losses in the yields of wheat, rice, maize, potato, soybean, and cotton (*Gossypium herbaceum* L.) [135]. To prevent productivity losses, agriculture must be able to deal efficiently with biotic stressors. Currently, chemical-based agricultural approaches are facing serious restraints due to health and environmental concerns. As a result, the demand for organically grown food has increased worldwide. In this scenario, PDS solutions, rich in karrikins and other bioactive compounds, could be a valued tool for alleviating biotic stresses in plants. The application of PDS solution increased ascorbic acid in carrots, which might help to control biotic stresses [111]. Biochar-derived smoke water exhibits repelling effects for nematodes and insects, and smoke water has potential to be used for the control of root-knot nematodes and olive fruit flies [136]. PDS solution enhanced flavonoid levels during in vitro seedling development in *Tulbaghia* species [106] and this increased flavonoid production might be related to defense against biotic stress. Later, the antibacterial and antifungal role of flavonoids was confirmed by Saini et al. [137], supporting their linkage with biotic stress management in plants. KARs are reported to enhance the antioxidant enzymes in the seed germination of teff (*Eragrostis tef* trotter.) [138], and antioxidants such as APX, CAT, POD, and SOD protect cell apoptosis by scavenging the ROS produced during biotic and abiotic stresses [139]. Beside these, SLs are reported to be involved in resisting attacks from pathogenic bacteria, fungi, nematodes, aphids, and pests, thus conferring biotic stress tolerance in plants. It inhibits the growth of invasive microorganisms, promotes stomatal closure, increases H_2_O_2_ production, and interacts with other defense hormones to enhance plant biotic stress tolerance [140]. SLs and KARs are both butenolide molecules that play essential roles in plant growth and development [141].

Several major biotic stresses in agriculture such as Fusarium wilt in banana, late blight in potato, bacterial blight in rice, and rust in wheat pose serious threats to global food production. Emerging research suggests that PDS solution may have potential in inducing resistance against such widespread pathogens by modulating signaling molecules like salicylic acid, jasmonic acid, and flavonoid biosynthesis pathways. The application of PDS in crops suffering from these stresses could support integrated pest and disease management programs in both organic and conventional agriculture systems.

Although meager data is available to validate the direct role of PDS in the control of biotic stress in plants, its potential in biotic stress management can be inferred by taking into account the antioxidant and antimicrobial properties of PDS, which induces defense responses in stressed plants. This potential of PDS may help plants in managing biotic stress by triggering defense mechanisms, increased resistance to pathogens and pests, and repelling pests, thus leading to stress tolerance. Furthermore, PDS could be integrated into in-practice pest management strategies for more viable and efficient solutions.

### 5.2. Flooding Stress Tolerance

Flooding is a serious environmental stress worldwide and is increasing in frequency due to changes in global climate [142]. Flooding is a destructive disaster that severely restricts agricultural production [143]. PDS solution enhanced stress tolerance in various plant species, including wheat, soybean, and rice. Growth restoration from PDS solution was observed in soybean under flooding and post-flooding stress [26,27]. Zhong et al. [27] found that stress mitigation by PDS solution involves the regulation of ornithine synthesis and the ubiquitin–proteasome pathway. Li et al. [26] revealed that the PDS solution supported soybean recovery by helping to balance sucrose and starch metabolism, enhance glycolysis, and boost the accumulation of proteins related to cell wall formation. In wheat, flooding reduced levels of RuBisCO activase and its large and small subunits, but applying PDS solution helped restore these important photosynthetic proteins [30]. The potential role of PDS in enhancing flood stress resolution should be further examined. Some key aspects include the recovery of chlorophyll *a* and *b*, glutamine, glutamic acid, aspartic acid, and serine contents by PDS solution in flooding-stressed plants [30]. Despite the significant output, further studies covering nutrient uptake and hormonal regulation in response to PDS are needed to uncover its potential benefits and limitations in flooding stress tolerance to improve crop resilience.

### 5.3. Salt Stress Tolerance

Soil salinization is one of the significant factors contributing to reduced crop yields worldwide [144,145]. This is caused by the accumulation of water-soluble salts above threshold levels within the soil layer, which adversely affects seedling growth and seed yield [146]. The application of PDS solution significantly alleviates salt stress by improving seed germination, seedling growth, and the regulation of antioxidant defense mechanisms in plants under salt stress. Priming with PDS solution resulted in improved seed germination under salt stress compared to salt-stressed seeds without PDS solution priming [105]. Rice seedlings treated with PDS solution showed increased activities of antioxidants and increased levels of K^+^ and Ca^2+^ while Na^+^ contents were decreased [24]. While talking about stress tolerance in salt-stressed plants, the Na^+^/K^+^ ratio is important as it indicates how well a plant is enduring under salt stress. Rice plants were reported to take up more Na^+^ than K^+^ when grown in various concentrations of salt, leading to a high Na^+^/K^+^ ratio. Alleviation through PDS solution restored a more balanced Na^+^/K^+^ ratio either by reducing sodium uptake or enhancing potassium uptake [24]. This increase in K^+^ and Ca^2+^ levels with a decrease in Na^+^ contents in rice seedlings sown from PDS solution-primed seeds compared to hydro-primed ones under saline conditions indicate the involvement of cell membrane transporter proteins adopting the exclusion mechanism for Na^+^. Later, these indications were confirmed by Komatsu et al. [31], who reported that most of the significantly changed proteins in PDS solution-treated wheat under salt stress were associated with protein metabolism and signal transduction in biological processes. It was observed that the increase in antioxidant activities in response to salt stress was regulated with the PDS solution application in maize [37]. The germination percentage of wheat seeds treated with salt stress was improved by the application of 2000 ppm PDS solution [38]. PDS solution-treated seedlings exhibited an improved activity of major antioxidative enzymes including POD, SOD, and APX under salt stress, subsequently leading to decreased levels of hydrogen peroxide and lipid peroxidase. More prominently, salt stress resulted in the changed expression of germination markers including *aPHY*, *TaSAM*, *TaBGU* (germination positive effectors), and *TaLEA* and *TaGARS34* (germination negative effectors). These changes at the genomic level suggest the involvement of PDS in stress tolerance via gene-level regulations. PDS solution was also found to modulate the transcript levels of several salt stress-responsive genes, including *TaSOS4*, *TaBADH*, and *TaHKT2*, further strengthening the concept of PDS solution-mediated stress tolerance [38]. These transcript-level regulations emphasize the complex molecular mechanisms responsible for the plant growth responses to PDS solution. Further research reflecting these insights is required to obtain more strategic solutions for plant growth improvement and stress tolerance.

Catav et al. [126] reported that the seedling length of wheat decreased by salt stress was recovered when 1000 ppm PDS solution was applied. It was demonstrated that in addition to modulating the physiological responses of salt-stressed wheat seedlings, PDS solution regulated the expression of some transcription factors and antioxidant enzyme-coding genes. PDS solution increased the expression levels of *TaDREB1*, *TaWRKY2*, *TaWRKY19*, *CAT*, and *Cu/Zn-SOD* in salt-treated seedlings. It was also documented that under normal growth conditions, PDS solution increased the transcription of *TaWRKY19* and *Cu/Zn-SOD* genes. Soybean treated with PDS solution developed better root growth under salinity stress [29]. Osmotin, alcohol dehydrogenase, and sucrose synthase increased with salt stress and decreased with additional PDS solution; however, H^+^ ATPase showed opposite effects in soybean and xyloglucan endotransglucosylase/hydrolase increased with salt and decreased with additional PDS solution [29]. APX increased with salt stress and decreased with additional PDS solution; however, H^+^-ATPase displayed opposite effects in wheat [31]. It is concluded that PDS significantly increased tolerance in plants when applied in the presence of salt stress.

The effects of PDS solution on Arabidopsis during salt stress have received more attention. According to research, exposure to smoke solutions can mitigate the harmful effects of salt by activating antioxidant defense systems, lowering oxidative damage, and enhancing seedling establishment in saline settings. The findings suggest that PDS solution exposure increases proline accumulation in Arabidopsis, which is an important osmo-protectant implicated in salt stress resistance [147]. PDS solution has also been reported to alter gene expression associated with stress tolerance mechanisms in Arabidopsis, indicating its potential as a natural growth enhancer under adverse conditions [148]. All these studies show PDS solution as a promising enhancement in salt stress tolerance based on the restoration of seed germination, plant growth, and regulation of stress-responsive genes in plants.

Salt stress has been shown to induce supplementary dormancy in seeds by modifying water uptake, hormone signaling, and gene expression associated with dormancy maintenance [149]. Recent studies demonstrate that salt-induced dormancy is associated with increased ABA production and decreased levels of important GA-responsive genes. Understanding how PDS solution interacts with salt stress pathways in Arabidopsis is critical to determine the potential uses of PDS solution-derived chemicals in increasing seed germination and plant resistance to stress.

### 5.4. Heavy Metal Stress Tolerance

Heavy metal stress has become a major concern in different terrestrial ecosystems worldwide. Extensive industrialization imparts detrimental effects on soil as well as on crop productivity by accumulating heavy metals [150]. Aslam et al. [22] documented an alleviation of CoCl_2_ in ipomea where the number of lateral roots, adventitious roots, and adventitious roots length of ipomoea cutting was restored, showing that PDS mitigated the negative effects CoCl_2_ in ipomea by boosting growth-related parameters [22]. A similar alleviating effect was observed in boron-stressed sorghum seedlings where an improvement in seed germination, seedling length/weight, and number of secondary roots was recorded [118]. This alleviation could be due to the involvement of PDS solutions in increasing the cell division process via enhanced food mobilization and decreasing the uptake of heavy metals through roots, thus finally resulting in alleviating the negative effects of stress on ipomoea and sorghum plants [22,118]. These results suggest that heavy metal stress can be reduced by the application of PDS solution.

PDS solution increased the resistance capacity of plants treated with different heavy metals. Rice roots were treated with PDS solution in the presence of Pb stress to determine Pb contents, electrolytes contents, total soluble sugar, total soluble protein, and antioxidant enzymes [39]. Pb and proline contents were reduced by PDS solution treatment, while the levels of Ca^2+^, K^+^, total soluble sugar, and total soluble proteins were raised in rice roots [39]. Mercury (Hg) and arsenic (As) stress significantly reduced photosynthetic pigments in wheat [127]. The application of PDS solution treatments ameliorated the drastic effects of Hg and As on the photosynthetic pigments in wheat leaves. PDS solution treatments restored rice and wheat seedling growth in the presence of Pb, Hg, and As stress and maintained optimum levels of CAT and APX [39,127]. Speciation is one of the main factors responsible for the toxicity and mobility of major heavy metals including Pb, Hg, Cd, As, and Cr. For instance, methylmercury is more toxic than elemental mercury and Cr that may be found either as Cr^3+^, a less toxic form, or in a highly toxic form as Cr^6+^. It is assumed that PDS solution may interfere with the speciation of heavy metals, thus changing their available forms to unavailable forms. This shift in speciation affects the mobility and availability of these heavy metals, making them less available to plants, thus resulting in less absorption. It could be inferred that PDS solutions may impart heavy metal tolerance to plants by inducing changes in metal speciation and activating antioxidant defense mechanisms. By modulating the speciation of heavy metals and improving the antioxidant capacity of plants, PDS solutions offer a promising strategy for improving plant resilience in heavy metal-affected areas. This approach shows potential for improving crop survival and productivity in metal-contaminated environments, leading to more economic and sustainable agricultural practices.

## 6. Conclusions

Smoke and its by-products are not new to agriculture, as they have been used in agriculture since ancient times. However, research into PDS solution and its stimulating effect on seed germination and post-germination stages has raised a new debate as to whether it should be taken only as a growth promoter or as a regulator with dual effects. We conclude that it is a biostimulant with a mixture of promoting and inhibitory effects in a concentrated form; however, PDS solution is confirmed as a significant promoting agent when used in diluted forms. A thorough analysis of recent studies suggests that the stimulatory or inhibitory properties of PDS are determined not by the actual concentrations of the stimulatory or inhibitory components, but by the balance between them.

Research on the significant role of PDS solution in coping and adapting to adverse environmental conditions has been noticeably expanded. In plants, both abiotic and biotic stress tolerance implies a series of complicated processes involving the regulation of different metabolites, genes, and proteins. Based on the different stress tolerance responses of plants induced by PDS solution, the current review offers a novel blend of several strategies adapted by plants at physiological, biochemical, genomic, and proteomic levels. PDS solution conferred stress tolerance by regulating mechanical stability, photosynthesis, and respiration at cellular levels. Various cellular phenomena including cell division, cell wall expansion, and cell membrane stability were enhanced by PDS application.

Plant response mechanisms to PDS solution also include the activation of different antioxidants, which in turn scavenge the ROS and revive the stomatal conductance and photosynthetic potential of plants. Molecular-level adaptations include gene expression changes with the regulation of stress-responsive genes (antioxidant defense, hormone signaling, and transcription factors) and the adjustment of hormone-associated genes and stress-responsive proteins. PDS solution application enhanced carbohydrate metabolism-related genes, aquaporins, redox homeostasis, transcription control, protein transport, proteins degradation, and signaling- and transport-related proteins in many plant species. These transcriptional- and translational-level changes are optimized by PDS solution to help plants efficiently adjust to various kinds of stresses.

PDS solution enhances nutrient uptake by strengthening root architecture and enriching soil microflora. All these phenomena help to elucidate the detailed mechanisms by which PDS solution alleviates various environmental stresses. Although this review managed to uproot various hidden approaches involving stress tolerance conferred by PDS solution to plants, a more detailed analysis of all metabolites and their genomic- and proteomic-level bases will be necessary for improved insight into the response mechanisms of plants to stresses arbitrated by PDS solution.

## Figures and Tables

**Table 1:** Effects of karrikins (KARs) and other components identified in PDS solution on seed germination and morphological and biochemical growth parameters of plants.

Solution Applied	Solution Concentrations	Seed Germination and Morphological and Biochemical Parameters Studied	Results	Ref.
Arabidopsis				
KAR	1 µM	Seed germination	Increased	[41]
KAR	1 µM	Seed germination, hypocotyl length, bud growth	Increased	[42]
KAR	1 µM	Seed germination, hypocotyl length	Increased	[43]
KAR	1 µM, 10 µM	Hypocotyl length	Increased	[44]
KAR	1 µM	Seed germination, hypocotyl length, cotyledon area, cotyledon petiole length	Increased	[45]
Lettuce				
KAR	10^−1^ µM	Seed germination	Increased	[32]
KAR	10^−1^ µM	α-amylase, biomolecules, lipase activity	Increased	[32]
Wild oat (*Avena sativa* L.)				
KAR	3000 µM	Seed germination, coat rupture, coleorhiza emergence, root emergence, water contents (%)	Increased	[46]
KAR	3 × 10^−3^ µM	ACC synthase, ACC oxidase	Increased	[46]
Cabbage (*Brassica oleracea* L.)				
KAR	3 × 10^−3^ µM	Seed germination, relative water contents	Increased	[47]
KAR	3 × 10^−3^ µM	SOD, CAT	Decreased	[47]
KAR	3 × 10^−3^ µM	Glutathione reductase	Increased	[47]
KAR	3 × 10^−3^ µM	*BOACS7*, *BOACS9*, *BOACS 11*, *BOACS2* genes	Increased	[47]
Redroot sage (*Salvia miltiorrhiza* Bunge)				
KAR	10^−3^ µM	Tanshinone I contents, NO contents, jasmonic acid contents	Increased	[48]
Brassica (*Brassica napus* L.)				
KAR	10^−1^ µM, 10^−2^ µM	Seed germination, mean germination index, germination rate	Increased	[49]
Lettuce				
Trimethyle butenolides	0.1 µM, 1 µM, 10 µM, 100 µM	Seed germination	Decreased	[50,51,52]
TMB	10^−1^ µM	Seed germination	Decreased	[32]
TMB	10^−1^ µM	α-amylase, biomolecules, lipase activity	Decreased	[32]
Hydroquinone	45 µM, 90 µM, 180 µM	Seed germination, seedling length	Increased	[8]
5,5-dimethyl-2(5H)-furanone	1 µM, 10 µM, 100 µM, 1000 µM	Seed germination	Increased	[53]
(5RS)-5-ethyl-2(5H)-furanone	1 µM, 10 µM, 100 µM, 1000 µM	Seed germination	Increased	[53]
Kangaroo paw (*Anigozanthos manglesii* Maund)				
Glyceronitrile	1 µM, 5 µM, 10 µM, 20 µM, 50 µM	Seed germination	Increased	[54]
Karoo Frayreed (*Rhodocoma arida* H.P.Linder & Vlok)				
Glyceronitrile	1 µM, 5 µM, 10 µM, 20 µM, 50 µM	Seed germination	Increased	[54]
Cat’s paw (*Anigozanthos humilis* Lindl.)				
Glyceronitrile	1 µM, 5 µM, 10 µM, 20 µM, 50 µM	Seed germination	Increased	[54]
Tobacco (*Nicotiana tabacum* L.)				
Catechol	10 µM	Root elongation, root hair elongation	Increased	[55]
Cucumber (*Cucumis sativus* L.)				
Catechol	50 µM	Phenolic contents	Increased	[56]
Lemongrass (*Cymbopogon flexuosus* Steud.)				
Catechol	5 µM	Seedling length, seedling weight	Increased	[57]
Catechol	5 µM	Essential oil contents, essential oil yield, total chlorophyll content, total carotenoid content	Increased	[57]
Catechol	5 µM	Proline contents	Decreased	[57]

**Table 2:** Effects of PDS solution and karrikins on seed germination of different plants.

Solution Applied	Solution Concentrations	Seed Germination (%)	Ref.
Chickpea			
PDS	2000 ppm	Increased	[25]
Cucumber			
PDS	5000, 10,000 ppm	Increased	[79]
Popcorn (*Sapium sebiferum* L. Roxb.)			
KAR1	0.001 µM	Increased	[80]
Aubergine (*Solanum melongena* L.)			
KAR	0.1 µM	Increased	[81]
Western Australian plants			
PDS	10, 20, 30, 40, 50%	Increased	[82]
Melon (*Cucumis melo* L.)			
KAR	0.1 µM	Increased	[83]
Snotblom (*Albuca pachychlamys* Baker), Blue squill (*Merwilla natalensis* Planch. Speta.), Society garlic (*Tulbaghia violacea* Harv.)			
PDS	1000, 2000 ppm	Increased	[84]
Hairy cup flower (*Angianthus tomentosus* J.C.Wendl.), Dwarf cup flower (Gnephosis tenuissima Cass.), Large cupper wire daisy (*Podolepis canescens* A.Cunn. ex DC.), Yellow buttons (*Myriocephalus guerinae* F.Muell.)			
KAR	0.00667 µM, 0.0667 µM, 0.6667 µM	Increased	[85]
Orobanche (*Orobanche minor* Sm.), Striga (*Striga hermonthica* Delile Benth.)			
KAR	1 × 10^−5^ µM–10 µM	Increased	[86]
Medicinal plants			
PDS	2000 ppm	Increased	[87]
Wallaby grass (*Austrodanthonia tenuior* Steud.), Bentham’s love-grass (*Eragrostis brownii* Kunth.), Paddock love-grass (*Eragrostis leptostachya* R.Br. Steud.), Hairy panic grass (*Panicum effusum* R.Br.), Two color panic grass (*Panicum simile* Domin.)			
PDS	10%	Increased	[88]
Beans (*Phaseolus vulgaris* L.)			
PDS	0.5, 1, 1.5, 2%	Increased	[89]

**Table 3:** Effects of PDS solution on morphological and physiological growth parameters of different plants.

Solution Applied	Solution Concentrations	Morphological, Physiological, and Biochemical Parameters Studied	Results	Ref.
Mung bean (*Vigna radiata* L.)				
PDS	1000, 2000 ppm	Roots initiation	Increased	[101]
Tomato (*Solanum lycopersicum* L.)				
KAR PDS	10^−4^ µM–0.1 µM KAR 2000 ppm PDS	Radical emergence, hypocotyle elongation, seedling weight Plant height, number of leaves, stem thickness, number of fruit per plant, mean fruit weight, plant biomass, mean fruit diameter	Increased	[102,103]
Rice				
PDS	1000, 2000 ppm	Seedling length, seedling weight	Increased	[16,104,105]
Wheat				
PDS	100, 1000 ppm	Seedling length, seedling weight	Increased	[17]
Maize				
PDS	400, 1600 ppm	Seedling length, seedling biomass	Increased	[37]
PDS	400, 1600 ppm	Chlorophyll *a*, chlorophyll *b*, carotenoids, total soluble proteins	Increased	[37]
Wild garlic (*Allium ursinum* L.)				
PDS	2000 ppm	Leaf length, root length, root number, seedling fresh biomass	Increased	[106]
PDS	2000 ppm	phenolic, flavonoid, condensed tannin	Increased	[106]
Ipomoea cuttings				
PDS	2000 ppm	Number of lateral roots, number of adventurous roots	increased	[22]
Maize				
PDS	2000 ppm	Seedling length, fresh and dry weight	Increased	[18]
PDS	2000 ppm	Photosynthetic pigments, carotenoids	Increased	[18]
PDS	1000, 2000 ppm	ABA contents	Decreased	[107]
Bentgrass (*Agrostis stolonifera* L.)				
KAR1	0.1 µM	Chlorophyll contents, POD, SOD, APX activities	Increased	[108]
Horticulture crops				
PDS	PDS obtained from different plants	Root length, shoot length, seedling fresh/dry weight, seedling vigor	Increased	[109]
PDS	PDS obtained from different plants	Ion contents, photosynthetic pigments, α-amylase activity, N, P, K contents	Increased	[109]
PDS	PDS obtained from different plants	ABA	Decreased	[109]
Grass pea (*Lathyrus sativus* L.)				
PDS	1, 5, 10, 20, 40%	Root length, shoot length, root dry weight, shoot dry weight, shoot width, number of branches, number of total nodule	Increased	[110]
PDS	1, 5, 10, 20, 40%	Shoot protein contents, K, P, shoot and root oxalyldiaminopropionic acid (OPAD)	Increased	[110]
Carrot (*Daucus carota* L.)				
PDS and KAR1	PDS treatments 25.8, 51.6, 103.2, 258.0 µg/L KAR1 treatments 0.00001 µM, 0.001 µM, 0.010 µM, 0.100 µM	Plant height, number of leaves, total leaf area, root length, root diameter, root fresh weight, root dry weight	Increased	[111]
PDS and KAR1	PDS treatments 25.8, 51.6, 103.2, 258.0 µg/L KAR1 treatments 0.00001 µM, 0.001 µM, 0.010 µM, 0.100 µM	Total chlorophyll, total carotenoids, chlorophyll fluorescence, intercellular CO_2_ concentration, stomatal conductance, net photosynthetic rate, beta carotene, ascorbic acid	Increased	[111]
Cabbage				
KAR1	10 µM, 0.1 µM, 0.001 µM	Root/shoot fresh and dry weight	Increased	[112]
KAR 1	10 µM, 0.1 µM, 0.001 µM	Photosynthetic rate, leaf relative water contents, water potential, leaf osmotic potential, membrane stability index, total chlorophyll, total sugar, total carotenoids, intercellular CO_2_ concentrations, transpiration rate	Increased	[112]
KAR 1	10 µM, 0.1 µM, 0.001 µM	Malondialdehyde (MDA), H_2_O_2,_ electrolyte leakage	Decreased	[112]
Wheat				
PDS	2000 ppm	Root/shoot length, seedling fresh weight	Increased	[17]
PDS	1000, 2000 ppm	Root/shoot length, seedling fresh weight	Increased	[38]
PDS	1000, 2000 ppm	H_2_O_2_, Thiobarbituric Acid Reactive Substances (TBARS)	Decreased	[38]
PDS	1000, 2000 ppm	APX, POD, SOD	Increased	[38]
PDS	2000, 4000 ppm	Seedling vigor, root length, leaf area, shoot length	Increased	[113]
PDS	2000, 4000 ppm	Water potential, relative water, chlorophyll *a*, chlorophyll *b*, total chlorophyll, membrane stability	Increased	[113]
Redroot sage				
PDS and KAR1	1000 ppm PDS 0.001 µM KAR	Biosynthesis of flavonoids and terpenoids	Increased	[114]

**Table 4:** Effects of PDS solution on morphological and biochemical growth parameters of different plants under stress.

Solution Applied	Solution Concentrations	Morphological and Biochemical Parameters Studied	Results	Ref.
Soybean				
PDS + NaCl	2000 ppm PDS + 100,000 µM NaCl	Main root length, total root fresh weight	Improved	[29]
Wheat				
PDS + Flooding	2000 ppm PDS + Flooding	Root length, leaf length, leaf weight	Improved	[30]
PDS + NaCl	2000 ppm PDS + 100,000 µM NaCl	Root length, root weight, leaf length, leaf weight	Improved	[31]
Maize				
PDS + TIBA	2000 ppm PDS + 24.78 µM TIBA	Plant height, leaf length, length of primary roots, number of secondary roots, root/shoot fresh weight	Improved	[98]
Rice				
PDS + Cadmium (Cd)	1000 ppm PDS + 100 µM Cd 1000 ppm PDS + 200 µM Cd 1000 ppm PDS + 400 µM Cd	Seedling length, fresh and dry weight	Improved	[98]
Pea				
PDS + SiO_2_ NPs	2000 ppm PDS + 665.6 µM SiO_2_ NPs	Seedling length, seedling fresh weight, number of leaves, number of secondary roots	Improved	[97]
Grapevine				
PDS + CdCl_2_	0.5% PDS + 54.5 µM CdCl_2_ 1% PDS + 54.5 µM CdCl_2_ 2% PDS + 54.5 µM CdCl_2_	Root and shoot length/fresh weight, number of leaves	Improved	[131]
Cabbage				
KAR + Cd	10 µM KAR1 + 44.5 µM Cd 0.1 µM KAR1 + 44.5 µM Cd 0.00001 µM KAR1 + 44.5 µM Cd	Seedling fresh/dry weight	Improved	[99]
Popcorn				
KAR1 + NaCl	0.001 µM KAR1 + 150,000 µM NaCl	Shoot length, survival rate, root length, lateral root length, fresh weight	Improved	[80]
KAR1 + Osmotic stress	0.001 µM KAR1 + 150,000 µM mannitol	Shoot length, survival rate, root length, lateral root length, fresh weight	Improved	[80]
Bentgrass				
KAR1 + Drought	0.1 µM KAR1 + Drought	Electrolyte leakage, MDA, CAT activity	Decreased	[108]
KAR1 + Drought	0.1 µM KAR1 + Drought	Relative water contents, chlorophyll, proline contents, APX, POD, SOD activities	Improved	[108]
KAR1 + Drought	0.1 µM KAR1 + Drought	*MAX2*, *KAI2*, *AFL1*, *ABF3*, *MYB13*, *DREB2A*, *Cu/Zn-SOD*, *APX2*, *CAT1*, *POD2* genes	Improved	[108]
KAR1 + Drought	0.1 µM KAR1 + Drought	*DLK2*, *KUF1*, *SMAX1*, *WRKY75*, *WRKY28*, *PPH*, *Chl-PRX* genes	Deceased	[108]
Grapevine				
PDS + CdCl_2_	0.5% PDS + 54.5 µM CdCl_2_ 1% PDS + 54.5 µM CdCl_2_ 2% PDS + 54.5 µM CdCl_2_	Chlorophyll, relative water, stomatal conductance, leaf temperature, total phenolics contents, SOD, APX, CAT activities, electrolyte leakages (%), proline, MDA	Improved	[131]
Wheat				
PDS + Flooding	2000 ppm PDS + Flooding	RuBisCO activase and RuBisCO large/small subunits, photosynthetic pigments, glutamine, glutamic acid, aspartic acid, serine	Improved	[30]
PDS + NaCl	2000 ppm PDS + 100,000 µM NaCl	APX, Ubiquitin, ATP	Decreased	[31]
PDS + NaCl	2000 ppm PDS + 100,000 µM NaCl	H^+^-ATPase	Improved	[31]
Soybean				
PDS + NaCl	2000 ppm PDS + 100,000 µM NaCl	Glycoproteins, H^+^ ATPase	Improved	[29]
PDS + NaCl	2000 ppm PDS + 100,000 µM NaCl	Osmotin, alcohol dehydrogenase, sucrose synthase, cellulose synthase, xyloglucan endotransglucosylase/hydrolase	Decreased	[29]
Rice				
PDS + Cd	1000 ppm PDS + 100 µM Cd, 1000 ppm PDS + 200 µM Cd 1000 ppm PDS + 400 µM Cd	Cell membrane stability, photosynthetic pigments, Na^+^, K^+^, Ca^++^	Improved	[99]
PDS + Cd	1000 ppm PDS + 100 µM Cd 1000 ppm PDS + 200 µM Cd 1000 ppm PDS + 400 µM Cd	Proline, total soluble sugar, POD, CAT, Cd	Decreased	[99]
Cabbage				
KAR + Cd	10 µM KAR1 + 44.5 µM Cd 0.01 µM KAR1 + 44.5 µM Cd 0.00001 µM KAR1 + 44.5 µM Cd	Water potential, leaf osmotic potential, membrane stability index, metal tolerance index, chlorophyll *a*, chlorophyll *b*, total chlorophyll, carotenoid, soluble sugars, intercellular CO_2_ concentrations, stomatal conductance, transpiration rate, DHAR, MDHAR, GST, CAT, GR, GPX, APX, SOD, POD, proline	Improved	[112]
KAR + Cd	10 µM KAR1 + 44.5 µM Cd 0.01 µM KAR1 + 44.5 µM Cd 0.00001 µM KAR1 + 44.5 µM Cd	Root Cd, shoot Cd, translocation factor, MDA, H_2_O_2_, electrolyte leakage	Decreased	[112]
Popcorn				
KAR1 + NaCl	0.001 µM KAR1 + 150,000 µM NaCl	Electrolyte leakage, MDA, H_2_O_2_, APX, POD	Improved	[80]
KAR1 + NaCl	0.001 µM KAR1 + 150,000 µM NaCl	SOD, CAT	Deceased	[80]
KAR + Osmotic stress	0.001 µM KAR1 + 200,000 µM mannitol	MDA, H_2_O_2_, SOD CAT, APX, POD, ABA	Decreased	[80]

## Data Availability

Not applicable.

## Abbreviations

The following abbreviations are used in this manuscript:

PDS	Plant-derived smoke
Karrikins	KARs
ROS	ROS
TMB	3,4,5-trimethyl-2(5H)-furanone or Trimethyle butenolides
ABA	Abscisic acid
GA	Gibberellic acid
